# Capgras syndrome and poor facial emotion recognition

**DOI:** 10.1192/j.eurpsy.2022.1989

**Published:** 2022-09-01

**Authors:** M. Zrelli, E. Bergaoui, N. Staali, M. Moalla, R. Lansari, A. Larnaout, W. Melki

**Affiliations:** 1 Razi Hospital, Psychiatry D, Manouba, Tunisia; 2 Razi Hospital, Psychiatry D, Tunis, Tunisia

**Keywords:** Capgras syndrome, face emotion recognition

## Abstract

**Introduction:**

Capgras syndrome is a disorder of personal identification characterized by the delusional belief that one or more persons close to the subject have been replaced by physically identical doubles.

**Objectives:**

To deepen our knowledge of the Capgras syndrome

**Methods:**

A case report about a Capgras syndrome.

**Results:**

We report the case of a 46-year-old female patient who was admitted in February 2021 for incoherent speech and behavior disorder against her family members. Three years ago, she started to have hypochondriacal concerns. A week before her admission, she threatened her husband with a knife and she was convinced that her daughters were dead and that they had been replaced by clowns. She had a Capgras delusion with a hallucinatory and interpretative mechanism against her daughters, she reported olfactory and cenesthetic hallucinations. Biological explorations and brain CT were normal. The PANNS scale showed a positive scale of 36, a negative scale of 39, the general psychopathology scale of 53. She had a total score of 30/40 on The Penn emotion recognition test. The patient had difficulty in recognizing low intensity emotions (0 or 1) and 50% of the false responses were for the neutral emotion with responses tending towards anger first and then sadness.

**Conclusions:**

Capgras syndrome remains poorly described in the literature and the hypotheses concerning its origin often oscillate between neuropsychological and psychodynamic. With the recent advances in neuroscience, it would be interesting to deepen its physiopathology in order to place it in a more modern nosological framework.

**Disclosure:**

No significant relationships.

